# Increased Expression of Alpha-, Beta-, and Gamma-Synucleins in Brainstem Regions of a Non-Human Primate Model of Parkinson’s Disease

**DOI:** 10.3390/ijms23158586

**Published:** 2022-08-02

**Authors:** Sandra Duperrier, Analia Bortolozzi, Véronique Sgambato

**Affiliations:** 1 Institut des Sciences Cognitives Marc Jeannerod (ISCMJ), Unité Mixte de Recherche 5229 du Centre National de la Recherche Scientifique (CNRS), 69675 Bron, France; sandra.duperrier@isc.cnrs.fr; 2 UFR Biosciences, Université Claude Bernard, Lyon 1, 69100 Villeurbanne, France; 3 Institut d’Investigacions Biomèdiques de Barcelona (IIBB), Spanish National Research Council (CSIC), August Pi i Sunyer Biomedical Research Institute (IDIBAPS), 08036 Barcelona, Spain; analia.bortolozzi@iibb.csic.es; 4 Biomedical Research Networking Center for Mental Health (CIBERSAM), Institute of Health Carlos III (ISCIII), 28029 Madrid, Spain

**Keywords:** Parkinson’s disease, alpha-synuclein, beta-synuclein, gamma-synuclein, MPTP, non-motor symptoms

## Abstract

Parkinson’s disease (PD) is characterized by cell loss in the substantia nigra and the presence of alpha-synuclein (α-syn)-containing neuronal Lewy bodies. While α-syn has received major interest in the pathogenesis of PD, the function of beta- and gamma-synucleins (β-syn and γ-syn, respectively) is not really known. Yet, these proteins are members of the same family and also concentrated in neuronal terminals. The current preclinical study investigated the expression levels of α-, β-, and γ-synucleins in brainstem regions involved in PD physiopathology. We analyzed synuclein expression in the substantia nigra, raphe nuclei, pedunculopontine nucleus, and locus coeruleus from control and parkinsonian (by MPTP) macaques. MPTP-intoxicated monkeys developed a more or less severe parkinsonian score and were sacrificed after a variable post-MPTP period ranging from 1 to 20 months. The expression of the three synucleins was increased in the substantia nigra after MPTP, and this increase correlates positively, although not very strongly, with cell loss and motor score and not with the time elapsed after intoxication. In the dorsal raphe nucleus, the expression of the three synucleins was also increased, but only α- and γ-Syn are linked to the motor score and associated cell loss. Finally, although no change in synuclein expression was demonstrated in the locus coeruleus after MPTP, we found increased expression levels of γ-Syn, which are only correlated with cell loss in the pedunculopontine nucleus. Altogether, our data suggest that these proteins may play a key role in brainstem regions and mesencephalic tegmentum. Given the involvement of these brain regions in non-motor symptoms of PD, these data also strengthen the relevance of the MPTP macaque model of PD, which exhibits pathological changes beyond nigral DA cell loss and α-synucleinopathy.

## 1. Introduction

Parkinson’s disease (PD) is the second most common neurodegenerative disease in the elderly. The clinical diagnosis of PD is based on the presence of bradykinesia, in combination with rest tremor, rigidity, or both, and on the validation of specific criteria [1]. At the anatomo-pathological level, the disease is characterized by the preferential and irreversible loss of substantia nigra dopaminergic (DA) neurons that project to the motor posterior putamen and by the presence of Lewy bodies and neurites in the remaining DA neurons [2]. It is accepted that the lesion of this DA nigrostriatal pathway is involved in the pathophysiology of bradykinesia, rigidity, and motor complications [3]. However, the pathological process of the disease in not limited to the dysfunction of the nigral DA system and involves several brainstem regions that exhibit Lewy bodies and/or neurites and cell loss as well [4,5,6,7]. There is a degeneration of serotonergic (5-HT) neurons issued from raphe nuclei [8,9,10] that is linked to several motor (tremor, dyskinesia) and non-motor (depression, apathy, and sleep problems) parkinsonian signs [11]. There is also a significant loss of cholinergic neurons from the pedunculopontine nucleus (PPN) in PD-affected brains [12,13]. This cell loss has been associated with PD symptom severity, especially akinesia and gait and balance deficits [13,14,15]. Finally, the PD pathological process also affects noradrenergic neurons from the locus coeruleus (LC) [7,16,17,18]. Noradrenergic deficits have been linked to cognitive dysfunction, REM sleep behavioral disorder, and freezing of gait [19,20].

More than 70 identified proteins have been discovered in Lewy bodies, among them alpha-synuclein (α-syn) [21,22,23]. The α-Syn protein has received major interest since mutations within its *SNCA* gene were identified as the first genetic cause of PD [24]. Additionally, it was subsequently discovered that multiplications of the *SNCA* gene cause PD [25,26,27]. α-syn is a protein of 140 amino acids, expressed in the central nervous system and particularly abundant in neurons at the synaptic terminals, reaching micromolar concentrations in synaptic boutons [28]. α-Syn is involved in various physiological functions [29]. It regulates synaptic vesicle trafficking, neurotransmitter exocytosis, and dopamine metabolism [30,31,32]. α-Syn also plays a role at the level of cell organelles maintaining mitochondrial and lysosomal homeostasis [33,34]. It interacts, for example, with mitochondrial or cytoskeletal proteins. In the nucleus, it can also bind to different regions of DNA to regulate the transcription of different genes [35,36]. The accumulation, misfolding, and aggregation of α-syn leads to the formation of large intracellular aggregates, the Lewy bodies. α-Syn is considered to be a potential biomarker of PD and more generally of synucleinopathies [37], disease heterogeneity among the synucleinopathies being caused by distinct α-syn strains [38]. α-Syn is toxic to the substantia nigra and causes the degeneration of DA neurons [39]. There is a negative correlation between the cell density in the substantia nigra pars compacta and the local α-syn charge in the substantia nigra of parkinsonian patients [40,41]. However, there is no relationship with disease severity or duration. Besides the substantia nigra, these α-syn deposits are present in many brain regions of PD patients, including brainstem nuclei, such as pedunculopontine and raphe nuclei and the locus coeruleus [4,5,6,42]. However, again, no correlations have been shown between the accumulation of α-Syn aggregates and disease severity or duration.

Other members of the synuclein family exist, in particular the proteins beta-synuclein (β-Syn) and gamma-synuclein (γ-Syn), which share substantial sequence homology with α-Syn and which are also present in the central nervous system and enriched in neuronal synaptic terminals [43]. As opposed to α-Syn, β-syn and γ-Syn are not found in Lewy pathology [44,45,46]. However, these proteins are quite intriguing as they are involved in the long-term regulation and maintenance of nerve terminal function and dopamine homeostasis and may compensate for each other. β-Syn physically interacts with DA and neurotoxic metabolites of DA, which can affect the aggregation propensities of β-Syn [47]. β-Syn potentiates synaptic vesicle dopamine uptake [48]. β-Syn is believed to act as a general synaptic chaperone [37]. It is able to inhibit α-syn aggregation and reduces α-syn toxicity [49] but is also able to induce neurotoxicity [50]. γ-Syn is also associated with neurodegeneration [51] and is involved in the modulation of DA transmission [52]. Finally, recent data indicate that β-Syn and γ-Syn are modulators of synaptic vesicle binding of α-Syn [53]. Interestingly, whether from post-mortem brain tissue [45,46,54] or cerebrospinal fluid [55] from patients with Lewy body diseases, studies that have investigated the expression of these synucleins show an overall increase in the brains of patients, including PD. However, if α-syn has clearly been singled out in the pathophysiology of PD, this is not the case for the other β- and γ-Syn proteins. Our goal was therefore to systematically analyze the expression of the three components of the synuclein family in different brainstem regions in an animal model of PD, the macaque monkey intoxicated with the neurotoxin 1-méthyl-4-phényl-1,2,3,6-tétrahydropyridine (MPTP). The potential link between synuclein expression levels and the severity of the parkinsonian score, the cell loss, or the time elapsed after MPTP was also investigated.

## 2. Results

### 2.1. Impact of MPTP Intoxication on Parkinsonism, DA Cell Loss, and Synuclein Levels in the SN

**Figure 1:**
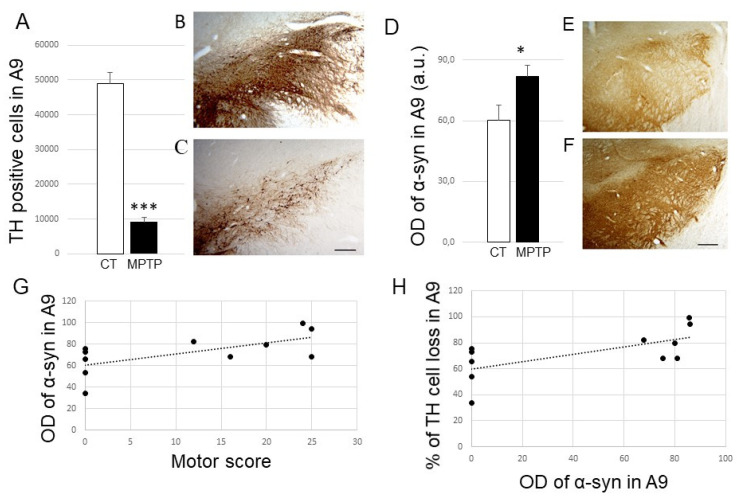
(**A**) Histogram represents the number of TH-positive cells in A9 from control (CT, *n* = 5) and MPTP-intoxicated (MPTP, *n* = 6) animals. *** *p* < 0.001 when compared to controls. (**B**,**C**) Photomicrographs at low magnification (×2.5) of coronal sections of a control (MF14) and an MPTP-intoxicated Macaca fascicularis (MF18), exemplifying TH labeling obtained at the level of the substantia nigra. Scale bar on panel C is 400 µm. (**D**) Histogram represents optical density of α-syn (in arbitrary units) in A9 from control (CT, *n* = 4) and MPTP-intoxicated (MPTP, *n* = 6) animals. * *p* < 0.05 when compared to controls. (**E**,**F**). Photomicrographs at low magnification (×2.5) of coronal sections of a control (MF15) and an MPTP-intoxicated (MF18) Macaca fascicularis, exemplifying α-syn labeling obtained at the level of the substantia nigra. Scale bar on F is 400 µm. (**G**,**H**) Positive correlation observed between the expression levels of α-syn (OD expressed in arbitrary units) in A9 and the maximal motor score (**G**) or the percentage of TH cell loss in A9 (**H**). Abbreviations: a.u, arbitrary units; OD, optical density; CT, control; TH, tyrosine hydroxylase.

As expected, the intoxication of animals with MPTP made it possible to induce parkinsonian symptoms in the animals. After MPTP, monkeys expressed a more or less severe parkinsonian motor score, ranging from 10 to 25 on a scale of 29 (Table 1). A significant loss of TH positive cells (*p* < 0.001) was detected in the substantia nigra (A9) of MPTP-intoxicated animals (on average 78%) (Table 1 and Figure 1A–C). Having MPTP monkeys that are heterogeneous in terms of motor score is an advantage when looking at whether something is related to the severity of the disease. For example, it is known that the greater the loss of dopaminergic neurons, the higher the score of the animal [56].

First, we were interested in the expression of α-syn in the substantia nigra (SN) in this animal model. We found that α-Syn expression levels were significantly increased in the SN from MPTP-treated monkeys (*p* < 0.05) (Figure 1D–F). Interestingly, we showed a positive correlation, although not very strong, between increased nigral α-Syn expression levels and parkinsonian score (R^2^ = 0.4172; *p* 0.03) (Figure 1G) or loss of TH-positive neurons in A9 (R^2^ = 0.4227; *p* value 0.0303; Figure 1H). We found no relationships between time after MPTP and TH or α-Syn expression levels in the SN.

**Figure 2:**
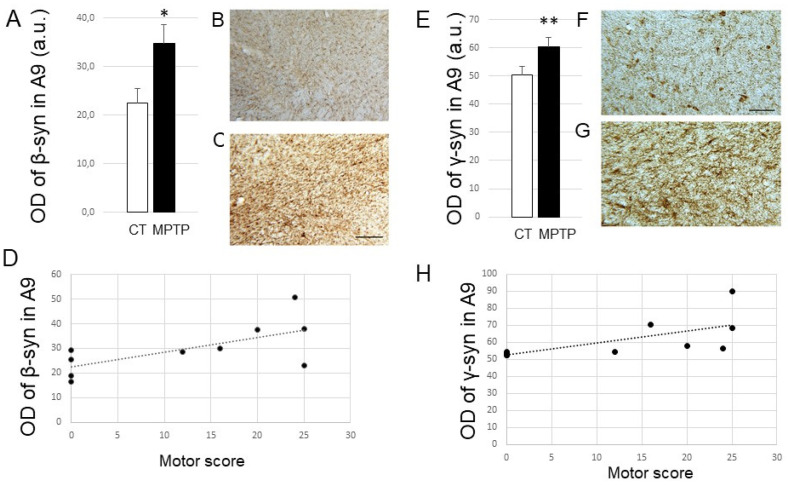
(**A**) Histogram represents optical density of β-syn (in arbitrary units) in A9 from control (CT, *n* = 5) and MPTP-intoxicated (MPTP, *n* = 6) animals. * *p* < 0.05 when compared to controls. (**B**,**C**) Photomicrographs at low magnification (×6.3) of coronal sections of a control (MF14) and an MPTP-intoxicated (MF18) Macaca fascicularis, exemplifying β-syn labeling obtained at the level of the substantia nigra. Scale bar on C is 200 µm. (**D**) Positive correlation observed between the expression levels of β-syn (OD expressed in arbitrary units) in A9 and the maximal motor score exhibited by the monkeys. (**E**) Histogram represents optical density of γ-syn (in arbitrary units) in A9 from control (CT, *n* = 5) and MPTP-intoxicated (MPTP, *n* = 6) animals. ** *p* <0.01 when compared to controls. (**F**,**G**) Photomicrographs at high magnification (×16) of coronal sections of a control (MF14) and an MPTP-intoxicated (MF18) Macaca fascicularis, exemplifying γ-syn labeling obtained at the level of the substantia nigra. Scale bar on F is 100 µm. (**H**) Positive correlation observed between the expression levels of γ-syn (OD expressed in arbitrary units) in A9 and the maximal motor score exhibited by the monkeys. Abbreviations: a.u, arbitrary units; OD, optical density; CT, control.

In view of these results, we then analyzed the expression of the other two synucleins in the SN from both control and MPTP-treated monkeys (Figure 2). Remarkably, β-Syn expression levels were also significantly increased in the SN from parkinsonian monkeys compared to control ones (*p* < 0.05; Figure 2A–C). Furthermore, a weak positive correlation was found between the nigral β-Syn expression levels and the severity of parkinsonism (Figure 2D; R^2^ = 0.4418 and *p* value of linear regression 0.036). β-Syn expression levels were also correlated with the DA nigral cell loss (R^2^ = 0,4159; *p* = 0.0441). Similarly, γ-Syn expression levels were also enhanced after MPTP (*p* < 0.01; Figure 2E–G) and correlated positively, although not very strongly, with the motor score (Figure 2H; R^2^ = 0.4594 and *p* value of linear regression 0.0219) and DA nigral cell loss (R^2^ = 0.3625; *p* = 0.05). Again, we found no correlations between time after MPTP and levels of β- or γ-Syn expression in the SN.

### 2.2. Impact of MPTP on Cell Loss in the DR, PPN, and LC

**Figure 3:**
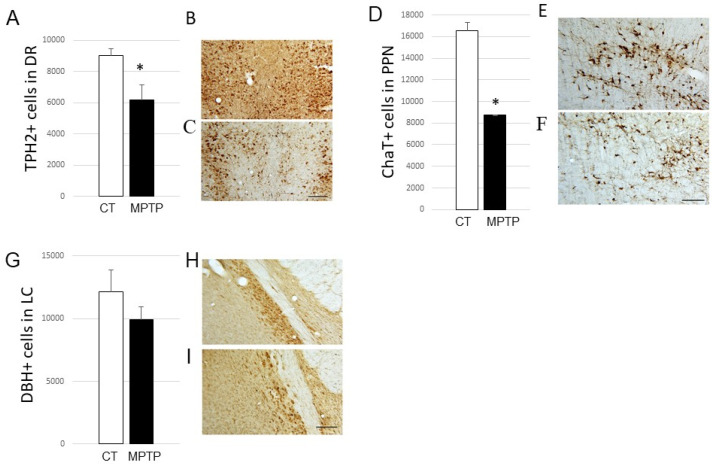
(**A**) Histogram represents the number of TPH2-positive cells in DR from control (CT, *n* = 5) and MPTP-intoxicated (MPTP, *n* = 6) animals. * *p* < 0.05 when compared to controls. (**B**,**C**) Photomicrographs at high magnification (×16) of coronal sections of a control (MF15) and an MPTP-intoxicated (MF18) Macaca fascicularis, exemplifying TPH2 labeling obtained at the level of the dorsal raphe. Scale bar on C is 100 µm. (**D**) Histogram represents the number of ChaT-positive cells in PPN from control (CT, *n* = 4) and MPTP-intoxicated (MPTP, *n* = 3) animals. * *p* <0.05 when compared to controls. (**E**,**F**) Photomicrographs at high magnification (×16) of coronal sections of a control (MF14) and an MPTP-intoxicated (MF16) Macaca fascicularis, exemplifying ChaT labeling obtained at the level of the pedunculopontine nucleus. Scale bar on F is 100 µm. (**G**) Histogram represents the number of DBH-positive cells in LC from control (CT, *n* = 3) and MPTP-intoxicated (MPTP, *n* = 3) animals. (**H**,**I**) Photomicrographs at high magnification (×16) of coronal sections of a control (MF24) and an MPTP-intoxicated (MF1) Macaca fascicularis, exemplifying DBH labeling obtained at the level of the locus coeruleus. Scale bar on I is 100 µm. Abbreviations: TPH2, tryptophan hydroxylase 2; DR, dorsal raphe; CT, control; ChaT, choline acetyl transferase; PPN, pedunculopontine nucleus; DBH, dopamine beta hydroxylase; LC, locus coeruleus.

On this neurotoxic monkey model of PD, we extended these experiments and examined the expression of different synucleins in three target regions: DR, PPN, and LC. First, we characterized the impact of MPTP treatment in these regions by counting neurons positively immunostained for TPH2 (i.e., serotonergic) in the DR, ChAT (i.e., cholinergic) in the PPN, and DBH (i.e., noradrenergic) in the LC (Figure 3).

Counts in the DR showed an impairment in the number of TPH2-positive cells in the DR from MPTP-treated monkeys (decrease reaching 31% in severely-lesioned monkeys; *p* < 0.05; Figure 3A–C). Regarding PPN, we found a 47% significant loss of ChaT-positive cells in MPTP-intoxicated monkeys (*p* < 0.05; Figure 3D–F). Finally, our data could not demonstrate any significant loss of DBH-positive neurons in the LC between the control and parkinsonian monkeys (Figure 3G–I).

### 2.3. Impact of MPTP Treatment on α-Synuclein Expression Levels in the DR, PPN, and LC

After characterizing the impact of MPTP intoxication on the different neuronal types of regions of interest other than the SN, we analyzed the expression levels of the three synuclein members.

For α-Syn, we found a significant increase in its expression levels in the DR from MPTP-treated monkeys compared to control ones (*p* < 0.01; see Supplementary Figure S1A–C). Interestingly, we found a positive correlation between this increase and the parkinsonian score (see Supplementary Figure S1D; R^2^ = 0.7965 and *p* value of linear regression 0.0002). Increased α-Syn expression levels were also correlated with the nigral DA cell loss (R^2^ = 0.7818; *p* = 0.0003) and with the raphe serotonergic loss (R^2^ = 0.4265; *p* = 0.0294). No relationships with post-intoxication time were found. On the other hand, disappointingly, we did not find significant changes in α-Syn levels in the PPN, nor in the LC (see Supplementary Figure S1E,F in Supplementary Material).

### 2.4. Impact of MPTP treatment on β- and γ-Synuclein expression levels in the DR, PPN, and LC

**Figure 4:**
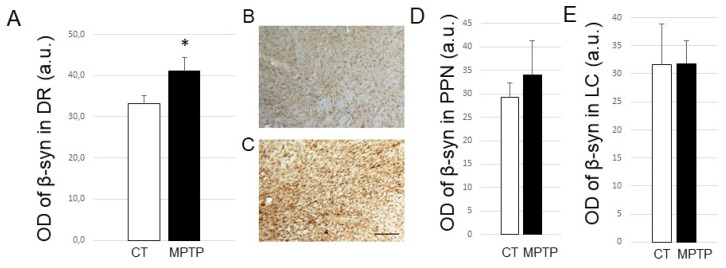
(**A**) Histogram represents optical density of β-syn (in arbitrary units) in DR from control (CT, *n* = 5) and MPTP-intoxicated (MPTP, *n* =6) animals. * *p* < 0.05 when compared to controls. (**B**,**C**) Photomicrographs at high magnification (×16) of coronal sections of a control (MF14) and an MPTP-intoxicated (MF18) Macaca fascicularis, exemplifying β-syn labeling obtained at the level of the dorsal raphe. Scale bar on C is 100 µm. (**D**) Histogram represents optical density of β-syn (in arbitrary units) in PPN from control (CT, *n* = 4) and MPTP-intoxicated (MPTP, *n* = 3) animals. (**E**) Histogram represents optical density of β-syn (in arbitrary units) in LC from control (CT, *n* = 3) and MPTP-intoxicated (MPTP, *n* = 3) animals. Abbreviations: a.u, arbitrary units; OD, optical density; DR, dorsal raphe; CT, control; LC, locus coeruleus.

As for β-Syn, we again found a significant increase in its expression levels in the DR from MPTP-treated monkeys compared to controls (*p* < 0.05; Figure 4A–C), but this increase could not be correlated with the neuronal loss in the DR nor the motor score. We also did not find significant changes in β-syn expression levels in the two other regions of interest, namely the PPN and the LC (Figure 4D,E).

**Figure 5:**
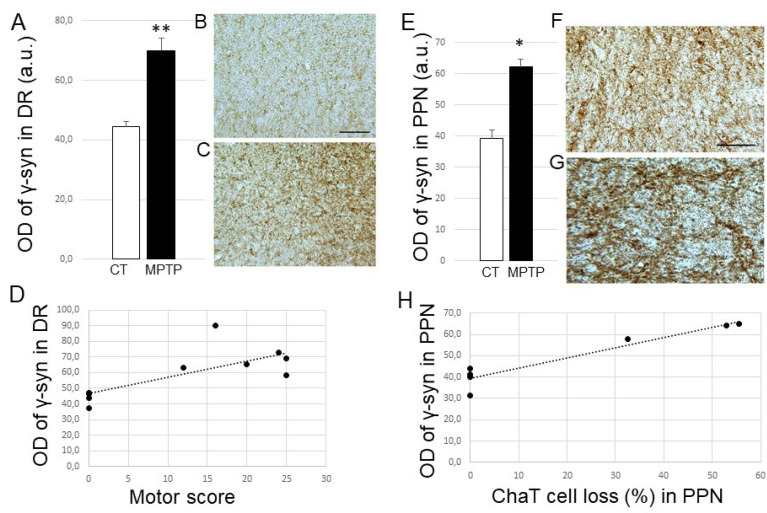
(**A**) Histogram represents optical density of γ-syn (in arbitrary units) in DR from control (CT, *n* = 5) and MPTP-intoxicated (MPTP, *n* = 6) animals. ** *p* < 0.01 when compared to controls. (**B**,**C**) Photomicrographs at high magnification (×16) of coronal sections of a control (MF14) and an MPTP-intoxicated (MF18) Macaca fascicularis, exemplifying γ-syn labeling obtained at the level of the dorsal raphe. Scale bar on B is 100 µm. (**D**) Positive correlation observed between the expression levels of γ-syn (OD expressed in arbitrary units) in DR and the maximal motor score exhibited by the monkeys. (**E**) Histogram represents optical density of γ-syn (in arbitrary units) in PPN from control (CT, *n* = 4) and MPTP-intoxicated (MPTP, *n* = 3) animals. * *p* < 0.05 when compared to controls. (**F**,**G**) Photomicrographs at high magnification (×16) of coronal sections of a control (MF14) and an MPTP-intoxicated (MF16) Macaca fascicularis, exemplifying γ-syn labeling obtained at the level of the pedunculopontine nucleus. Scale bar on F is 100 µm. (**H**). Positive correlation observed between the expression levels of γ-syn (OD expressed in arbitrary units) and the percentage of ChaT cell loss in PPN. Abbreviations: a.u, arbitrary units; OD, optical density; DR, dorsal raphe; CT, control; PPN, pedunculopontine nucleus.

Finally, for γ-Syn, we were able to demonstrate a significant increase in its expression levels in the DR from parkinsonian monkeys compared to controls (*p* < 0.01; Figure 5A–C). A positive correlation was found between this increase and the animals’ motor score (Figure 5D; R^2^ = 0.5735, *p* = 0.007) but not the serotonergic raphe loss (not shown). On the other hand, we also found an increase in the expression of γ-Syn levels in the PPN from parkinsonian monkeys compared to control ones (*p* < 0.05) (Figure 5E–G), and this increase could be positively correlated with the loss of cholinergic neurons detected in the PPN of the MPTP-treated animals (Figure 5H; R^2^ = 0.9034, *p* value of linear regression 0.001). Increased γ-Syn levels within the PPN were correlated with the motor score (R^2^ = 0.8894; *p* = 0.0014). Finally, no modification of γ-Syn expression could be detected in the LC (see Supplementary Figure S1G). Here, again, the time elapsed after MPTP had no impact on γ-Syn expression levels.

## 3. Discussion

Several important findings emerge from this study performed on the MPTP-intoxicated macaque model of PD. First, in the dopaminergic-depleted SN, all three synucleins have an increased expression that correlates, in a more or less strong way, with the severity of the nigrostriatal lesion and parkinsonian symptoms. Second, in the DR, the three synucleins are enhanced, but only α- and γ-Syn are linked to the motor score and associated cell loss. Third, in the PPN, only increased γ-Syn expression levels are detected after MPTP, but they correlate with the motor score and the cholinergic cell loss. Finally, there are no variations in synuclein expression in the LC, for which we did not detect a significant noradrenergic cell loss after MPTP intoxication.

The nigral increase in α-Syn is not new, as it has already been shown in parkinsonian patients and PD animal models. In PD patients, a correlation study indicated a close relationship among decreased TH immunoreactivity, α-Syn accumulation, and neuronal loss [40]. Another study showed a negative correlation between cell density in the SN pars compacta and the local load of α-Syn, but there were no relationships shown with disease duration or Hoehn and Yahr stage [41]. Several animal studies have shown an increase in α-Syn or its phosphorylated form in the SN pars compacta in aged and/or MPTP-treated monkeys [39,57,58,59,60]. These studies have shown correlations between increased α-Syn levels and a decreased number of cells in the SN. We confirm a relationship, although not very strong, between nigral α-Syn levels and TH loss (the more severe the TH loss, the stronger the increase in α-Syn) but also extend these results by showing that the more severe the monkey, the greater the increase in α-Syn.

MPTP is a neurotoxin that inhibits complex I of the mitochondrial respiratory chain through its active metabolite, MPP+, which impairs ATP production and increases oxidative stress, ultimately inducing cell degeneration. MPTP induces the lesion of dopaminergic neurons and accumulation of α-Syn in the SN in non-human primates [57,59,61,62,63,64] and in mice [65]. The MPTP-induced increase in α-Syn is neuronal (59,63,64] and does not affect astrocytes or microglia [63]. MPTP has been shown to cause the redistribution of α-Syn from axons to cell bodies and dendrites [61,62,63]. There is also the accumulation of α-Syn in enlarged dystrophic axons [63] and in GABAergic fibers in the SN [66]. In mice treated with multiple injections of MPTP over several days, the loss of dopaminergic cells in the SN is achieved within a few days, and α-Syn upregulation reaches its peak during the first week after intoxication [65]. In monkeys receiving a single injection of MPTP, neuronal injury occurs over several weeks, and α-Syn expression is still elevated until several months after intoxication [62,66]. In monkeys receiving multiple injections, there is an increase in α-Syn immunoreactivity that is detected only 10 days after the onset of intoxication [61], but also several years later, in the remaining neurons [57]. Our results are in agreement with these studies and show that the increased nigral levels of α-Syn reflect the severity of the MPTP-induced injury and not the time after intoxication. Taken together, these data emphasize that MPTP can induce long-lasting changes in α-Syn expression and that α-Syn expression and the degenerative process are closely linked. Moreover, α-Syn levels also increase with normal aging in primate SN [39,58]. Thus, there is a strong link between the normal (aging) or induced (by MPTP) neurodegenerative process and the α-Syn response. Finally, the use of MPTP in monkeys does not result in Lewy body formation as in humans. One research team wondered whether the absence of Lewy bodies was due to the experimental post-MPTP intoxication time, which may be too short compared to the time scale necessary for Lewy body formation in humans (around 10 years). However, this team failed to detect Lewy bodies in brain tissue from monkeys with stable parkinsonian symptoms at least 10 years after MPTP intoxication, despite consistent nigral degeneration and increased alpha-syn in the remaining neurons [57].

In parallel with the increase in α-Syn in the SN, we also show an increase in the nigral expression of β-Syn and γ-Syn, which is also related, although not very strongly, to the parkinsonian score. These findings indicate that both synucleins are sensitive to dopaminergic denervation driven by MPTP and therefore associated with DA loss and parkinsonian deficits. However, there are few preclinical studies focusing on β- and γ-Synucleins in the DA system. Interestingly, one study has shown that β-Syn confers resistance to MPTP in mice [48], and another one found that mice lacking β-Syn have deficits in sensorimotor coordination [67]. These data demonstrate that β-Syn is crucial in the regulation of dopamine in nigrostriatal neurons and concomitant motor function. Regarding γ-Syn, its inactivation in mice affects non-motor symptoms (psycho-emotional status and cognitive abilities) and DA transmission [68,69], while its overexpression induces loss of spinal motor neurons [51]. In agreement, the overexpression of γ-Syn in mouse DA neurons mainly reduces nigrostriatal DA neurotransmission and evokes motor and cognitive deficits [52]. At the clinical level, somewhat contradictory data were obtained. A study performed by mass spectrometry has shown that β-Syn concentrations in cerebrospinal fluid are not different between control and parkinsonian subjects, but slightly higher in demented parkinsonian patients [55]. Another study performed on post-mortem samples by RT-PCR has shown elevated β-Syn transcripts in caudate from PD patients [54]. In addition, post-mortem hippocampal accumulations of β-Syn and γ-Syn were evidenced in PD patients [45]. Additionally, oxidized γ-Syn (colocalized with phosphorylated α-syn) was found in Lewy bodies in the amygdala and substantia nigra of PD patients [46]. Taken together, these data suggest that in addition to α-Syn, β- and γ-Syn might be potential markers of motor and non-motor symptoms.

The results obtained within the dorsal raphe are also very interesting, as we show for the first time an increase in the three synuclein members after MPTP intoxication. The increase in α-Syn in the raphe of parkinsonian patients is well known [4,6,42], but the fact that the other synuclein members are increased in the DR of a PD animal model is totally new. We did not find any correlation between this increase and the degenerative state of 5-HT cells. However, it is important to know that the loss of 5-HT somas is dependent on the intoxication parameters used with MPTP, monkeys moderately damaged not necessarily exhibiting a loss of TPH2-positive cells in DR contrary to severely lesioned ones [56,70,71]. Instead, we found a link between α-Syn or γ-Syn (not β-Syn) with the motor score. The expression of these two synuclein members is therefore sensitive to the impact of MPTP both on the lesion it causes and on the severity of parkinsonism it induces. Interestingly, the viral overexpression of α-Syn in raphe 5-HT neurons of rodents leads to serotonergic dysfunction and an anxious- and depressive-like phenotype [72,73]. At the clinical level, the raphe α-Syn increase has never been linked to the severity or duration of the disease. It is most certainly due to the clinical heterogeneity of the patients and the inherent difficulty in classifying them. However, it is known that degeneration of 5-HT neurons is linked to several motor (tremor, dyskinesia) and non-motor (depression, apathy, and sleep problems) parkinsonian signs [11]. Finally, the fact that γ-Syn raphe increases correlate positively with the motor score suggests that γ-Syn might be a better marker of disease severity compared to β-Syn. It would be very interesting to further investigate the impact of γ-Syn modulation on the integrity of the 5-HT system and parkinsonian symptomatology.

In parallel to the SN and DR, which are the two regions in which we were expecting modifications of synuclein expression levels, there are other important regions, the PPN and the LC, which are involved in the pathophysiology of PD. There is a significant loss of cholinergic neurons from the PPN in PD-affected brains [12,13]. Whether this loss is a downstream response to prior α-Syn pathology and the death of monoaminergic neurons, or whether the death of PPN cholinergic neurons precipitates the loss of DA neurons [74,75], is unknown. However, it is associated with PD symptom severity, especially akinesia and gait and balance deficits [13,14,15]. Noradrenergic deficits from the LC also occur in PD [7,17,18,76] and have been linked to cognitive dysfunction, REM sleep behavioral disorder, and freezing of gait [19,20]. In addition, studies have shown that cholinergic neurons in the PPN and NA neurons in the LC may be affected in MPTP-intoxicated monkeys. It was therefore highly relevant to examine the expression of the three synuclein members in these two other nuclei on our animal model. We were able to show a very clear cholinergic deficit (47%) in the PPN but no cell loss in the LC, indicating a limitation of our MPTP model, which cannot reproduce all the anatomo-pathological features present in humans. Regarding PPN, our data agree with a previous study evidencing a 30% loss of cholinergic neurons in aged monkeys intoxicated with MPTP [77]. We could not evidence altered levels of α- or β-Syn but found an increased γ-Syn expression, which was negatively linked to the cholinergic cell loss. These results strength the possibility that γ-Syn might be a better marker of disease compared to the other synucleins, although it cannot be excluded that the lack of change in α-Syn (and β-Syn) levels in the PPN might be due to the small number of animals intoxicated with MPTP or to the rapid neurotoxic action of MPTP and the early evolution of parkinsonism it induces, in contrast to the mechanisms of long-term neurotoxicity that may take place in humans.

Finally, we did not obtain a significant cell loss in the LC (despite an 18% reduction) after MPTP, and consequently, we observed no significant changes in synuclein expression levels. Here, again, the lack of increased α-Syn (and β-Syn) levels in the LC may be specifically related to the neurotoxin MPTP (i.e., rapid intoxication protocol and early evolution of parkinsonism). This is clearly an important limitation of our animal model, since in PD patients, several postmortem pathological studies have highlighted a loss of NA neurons in the LC, which would be as important as that of DA neurons in the SN [5,7,16,76,78,79,80,81,82,83]. However, in monkeys intoxicated with MPTP, the data are much less consensual, with very variable results from one study to another, highlighting that the parameters used for MPTP intoxication have an important impact on the damage, or lack thereof, to NA neurons from the LC [71]. Indeed, while acute MPTP protocols fail to induce significant LC cell loss [84,85], chronic ones exhibit prominent LC damage [86,87]. As our monkeys were intoxicated with MPTP over a short period of time, our results are consistent with an MPTP regimen which does not allow for NA insult within the LC. Furthermore, NE terminals in projection regions might degenerate more importantly than cell bodies within the LC [88,89,90]. Further experiments would be necessary to clearly assess synuclein expression in a PD monkey model exhibiting significant LC cell loss.

To conclude, there are very few data in the literature evaluating the levels of β- and γ-Syn in the brainstem, and those that do exist are generally described as a compensatory mechanism associated with α-Syn-mediated pathology, although one must be careful in this interpretation. In this study, the data clearly seem to indicate that the brain areas most vulnerable to MPTP are SN>DR-PPN>LC in the present monkey model (Table 2).

Within these nuclei, MPTP treatment mostly altered α-Syn and γ-Syn expression levels, and to a lesser extent β-Syn ones, suggesting that the three components of the synuclein family could regulate monoaminergic transmission. Further studies will be needed to investigate the impact of modulating these synucleins in these regions on monoaminergic transmission and behavior.

## 4. Materials and Methods

### 4.1. Ethical Statement

All studies were carried out in accordance with European Communities Council Directive of 2010 (2010/63/UE) as well as the recommendations of the French National Committee (2013/113). There were also approved by the local ethical committee CELYNE C2EA #42 (05/18/2011 and 10/10/2017).

### 4.2. Animals

Two groups of adult male macaque monkeys were used in this study: control (untreated) monkeys (*n* = 5) and parkinsonian monkeys (i.e., intoxicated with the neurotoxin 1-methyl 4-phenyl 1,2,3,6-tetrahydropyridine (MPTP)) (*n* = 9). Taking into consideration the three Rs (Reduction, Refinement, and Replacement) for animal experimentation, we used behavioral data and brain tissue from monkeys previously used [56,74]. All monkeys weighed between 4 and 6 kg and were aged between 3 and 5 years. They were kept under standard conditions (12 h light cycles, 23 °C, and 50% humidity).

### 4.3. MPTP Intoxication

Nine monkeys (*Macaca fascicularis* MI 81, MI 93, MF1, MF4, MF11, MF16, MF17, MF18, and MF25) were rendered parkinsonian by intoxication with 1-methyl 4-phenyl 1,2,3,6-tetrahydropyridine (MPTP) (Table 1). Briefly, the monkeys received 3 to 4 intramuscular injections of MPTP (Sigma-Aldrich, Saint-Quentin-Fallavier, France) at a dose of 0.4 mg/kg under light anesthesia (ketamine 0.5 mg/kg, atropine 0.05 mg/kg) until the emergence of parkinsonian symptoms. MPTP intoxication was stopped once most of the motor parkinsonian symptoms had appeared. The control monkeys (*n* = 5) received no injections and were therefore not lesioned.

### 4.4. Parkinsonism Assessment

The severity of parkinsonian symptoms induced by MPTP treatment was assessed longitudinally using the rating scale proposed by Schneider and Kovelowski (1990) as previously described [56]. This scale includes 12 items rated between 0 (normal or none) and 2 or 3, with a total score of 29. It considers classical motor symptoms (bradykinesia, rigidity, tremor, freezing, posture, and arm posture), but also spontaneous activities (arm movements, spontaneous eye movements, and home cage activity) and other activities (vocalization, triggered eye movements, and feeding). For each item, scored from 0 to 2 or from 0 to 3, the higher the score, the more symptoms or deficits the animal has (the minimum score 0 meaning normal or none, the maximum score (2 or 3 depending on the item) indicating marked, severe, or absent). The higher the total score (out of 29), the more symptomatic the monkey (see Table 1).

### 4.5. Immunohistochemistry

#### 4.5.1. Tissue Preparation

At the time of the sacrifice, the animals were deeply anesthetized (ketamine at 1 mg/kg followed by a lethal dose of pentobarbital) and perfused transcardially with 400 mL of saline (0.9% at 4 °C) followed by 5 l of 4% paraformaldehyde (Sigma-Aldrich, Saint-Quentin-Fallavier, France) [in 0.1 M phosphate-buffered saline (PBS), pH 7.4 at 4 °C] and 1 l of PBS with 5% sucrose. Because we have considered the 3 Rs rule (see Animals section above), MPTP-treated animals involved in this study were sacrificed after a variable time ranging from 1 to 20 months after intoxication, and the potential impact of this factor on synuclein expression levels was subsequently analyzed. After perfusion, the brains were removed from the skull, rinsed in PBS complemented with 10% sucrose for 1 day and 20% sucrose for one further day, and then frozen and cut into 50 µm-thick sections coronally on a freezing microtome. Free-floating sections were conserved at –20 °C in a cryoprotective solution containing 30% ethylene glycol, 30% glycerol, and 0.25 M Tris buffer (Sigma-Aldrich, Saint-Quentin-Fallavier, France) until they were processed for immunohistochemistry. It should be noted that the SN and DR could be collected from all brains of monkeys. On the other hand, brain sections passing through the PPN and LC were recovered from 3–4 controls but from only 3 MPTP-treated monkeys.

#### 4.5.2. Immunostaining

Free-floating adjacent sections were rinsed in Tris-buffered saline (TBS; 0.25 M Tris and 0.25 M NaCl, pH 7.4), incubated for 10 min in TBS containing 3% H_2_O_2_ and 10% methanol, and then rinsed three times for 10 min each in TBS. After 30 min incubation in 0.2% Triton^TM^ X-100 in TBS, the sections were rinsed three times in TBS, saturated for 30 min in 30% goat serum in TBS, and rewashed. These were then incubated for 72 h at 4 °C with the following primary antibodies: anti-α-syn 1/2000 rabbit monoclonal from Abcam (Abcam, Paris, France) (MJFR1, catalog number ab138501), anti-β-syn 1/100 mouse monoclonal from Merck (Merck, Molsheim, France) (Syn207, catalog number 36-009), and anti-γ-syn 1/2000 rabbit polyclonal from Abcam (catalog number ab55424). Depending on the region of analysis considered (substantia nigra, dorsal raphe nucleus, pedunculopontine nucleus, or locus coeruleus), adjacent sections to those used for synuclein detection were additionally stained according to the same protocol with the following antibodies: anti-tyrosine hydroxylase (TH) 1/5000 mouse monoclonal from Euromedex (Euromedex, Souffelweyersheim, France) (catalog number 22941), anti-tryptophan hydroxylase 2 (TPH2) 1/800 sheep polyclonal from Millipore (Merck Millipore, Molsheim, France) (catalog number ab1541), anti-choline acetyl transferase (ChaT) 1/200 goat polyclonal from Millipore (catalog number ab144P), or anti-Dopamine beta hydroxylase (DBH) 1/2000 rabbit polyclonal from Millipore (catalog number ab1585). After three rinses in TBS, the tissue sections were then incubated for 2 h at room temperature with the corresponding secondary biotinylated antibody (1/500 from Abcys; Eurobio Scientific, Les Ulis, France) in TBS. After being washed, the sections were incubated for 90 min at room temperature in the Vectastain Elite ABC Peroxydase kit (final dilution, 1/50; Abcys; Eurobio Scientific, Les Ulis, France). The sections were then rinsed twice in TBS and twice in Tris buffer (0.25 M Tris, pH 7.4) for 10 min each, placed in a solution of H_2_O containing 0.1% 3,3′-diaminobenzidine (DAB; 50 mg/100 mL), and developed by H_2_O_2_ (0.02%). The specificity of the immunostaining was assessed by omission of the primary antibody from the protocol. After processing, the tissue sections were mounted onto gelatin–alum-coated slides and dehydrated through graded alcohol to xylene for light microscopic examination using a computerized image analyzer (Mercator, ExploraNova, La Rochelle, France). 

### 4.6. Soma Quantification

Soma quantification was performed under blinded conditions relative to the animal using non-stereological but computer-assisted counting, as in our previous studies [56,91,92,93]. For each monkey, stained cells for TH (in SN), TPH2 (in raphe nuclei) [and ChaT (in PPN), and DBH (in LC) when possible (see tissue preparation)] were plotted at x16 magnification and counted after cartography of the different brain regions. TH-positive cells were counted on nine regularly spaced sections encompassing A8 (peri and retrorubral area), A9 (pars compacta), and A10 (ventral tegmental area) dopamine regions, as previously described [56]. The distribution of TPH2-positive cells was examined throughout five regularly spaced sections covering the antero-posterior extent of raphe nuclei (as previously described in [56]). Positive cells for ChaT were counted on four regularly spaced sections covering the antero-posterior extent of the pedunculopontine nucleus. Finally, positive cells for DBH were counted on six regularly spaced sections covering the antero-posterior extent of the locus coeruleus. For each marker, the total number of labelled cells was estimated after correction by the Abercrombie method, as previously described [56].

### 4.7. Optical Density Measurements 

α, β, and γ-Synuclein expression levels were analyzed under blinded conditions relative to the animal by optical density measurements using Image J software. For the SN (identified by the labeling of TH, the dopamine synthesis enzyme), the expression of each synuclein was measured on five sections distributed along the rostro-caudal axis of the region. For the DR (identified by the labeling of TPH2, the serotonin synthesis enzyme), the expression of each synuclein was measured on three sections distributed along the rostro-caudal axis of the region. For the PPN (detected by the labeling of ChaT, which marks the cholinergic neurons of the PPN), the expression of each synuclein was measured on 3–4 sections distributed in the rostro-caudal axis of the region. Finally, for the LC (detected by the labeling of DBH, which is the noradrenaline synthesis enzyme), the expression of each synuclein was measured on 3 sections distributed in the rostro-caudal axis of the region.

### 4.8. Statistical Analysis

All statistical analyses were performed using GraphPad Prism software. Immunohistochemical data were analyzed using non-parametric Mann–Whitney tests with *p* < 0.05. Histograms represent mean ± SEM. Linear regressions were tested using GraphPad Prism as well.

## Figures and Tables

**Table 1:** Table 1 indicates the maximal parkinsonian score and the percentage of DA cell loss in the substantia nigra pars compacta (A9) obtained for each MPTP-intoxicated monkey.

MPTP-Intoxicated Monkeys	Maximal Motor Score (on 29)	TH Cell Loss in A9 (%)
MF1	21	77.5
MF4	16	75.4
MF11	12	67.8
MF16	25	86
MF17	25	81.1
MF18	24	85.6
MF25	20	79.9
MI 81	12	75
MI 93	10	73

**Table 2:** Table 2 summarizes the main results obtained in the different regions of interest for the cell loss analysis, the levels of the different synucleins, and the correlation analysis in the MPTP-intoxicated monkeys compared to the control group. Abbreviations: SNc, substantia nigra pars compacta; DR, dorsal raphe; PPN, pedunculopontine nucleus; LC, locus coeruleus; NA, not applicable.

Brain Regions	Cell Loss	Alpha-Synuclein Levels	Correlation Motor Score	Correlation Cell Loss
SNc	DA *p* < 0.001	*p* < 0.05	Yes *p* = 0.033	Yes *p* = 0.0303
DR	5-HT *p* < 0.05	*p* < 0.01	Yes *p* = 0.0002	Yes *p* = 0.0003
PPN	ChaT *p* < 0.05	unchanged	NA	NA
LC	unchanged	unchanged	NA	NA
	**Cell Loss**	**Beta-Synuclein Levels**	**Correlation Motor Score**	**Correlation Cell Loss**
SNc	DA *p* < 0.001	*p* < 0.05	Yes *p* = 0.036	Yes *p* = 0.0441
DR	5-HT *p* < 0.05	*p* < 0.05	No correlation	No correlation
PPN	ChaT *p* < 0.05	unchanged	NA	NA
LC	unchanged	unchanged	NA	NA
	**Cell Loss**	**Gamma-Synuclein Levels**	**Correlation Motor Score**	**Correlation Cell Loss**
SNc	DA *p* < 0.001	*p* < 0.01	Yes *p* = 0.0219	Yes *p* = 0.05
DR	5-HT *p* < 0.05	*p* < 0.01	Yes *p* = 0.007	Yes *p* = 0.0011
PPN	ChaT *p* < 0.05	*p* < 0.05	Yes *p* = 0.0014	Yes *p* = 0.001
LC	unchanged	unchanged	NA	NA

## Data Availability

The data supporting the findings of the study are available from the corresponding author upon request.

## Supplementary Materials

Supplementary materials can be found at https://www.mdpi.com/article/10.3390/ijms23158586/s1.
