# Supplementary figures and images for: Increased Expression of Alpha-, Beta-, and Gamma-Synucleins in Brainstem Regions of a Non-Human Primate Model of Parkinson’s Disease
Source: Int J Mol Sci. 2022 Aug 2;23(15):8586. doi: 10.3390/ijms23158586 (PMC9369189; doi:10.3390/ijms23158586)

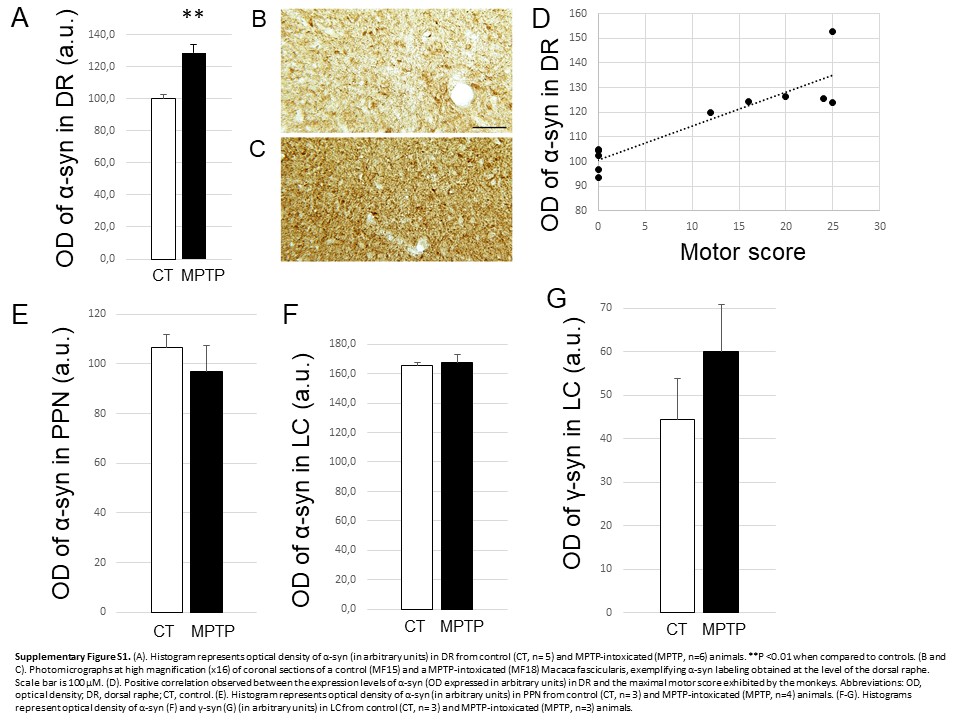

Supplement: Supplementary file 1 [file ijms-23-08586-s001.zip › ijms-1847031-supplementary.jpg]
